# Intermittent Fever-Cases

**Published:** 1881-10

**Authors:** E. W. Rushmore

**Affiliations:** Plainfield, N. J.


### CLINICAL BUREAU.

### INTERMITTENT FEVER.

*Hellebore, Ars., Rhus, Natr. mur.*

E. W. Rushmore, M. D., Plainfield, N. J.

(*Read before N. J. State Homœopathic Medical Society, at Newark, May,* 1881.)

*Helleborus.*—August 23d, 1879. Mr.—— has been attacked for three days, at 7.30 P. M., with shivering, heat and sweating at same time, without coldness, lasting several hours. With the first attack, pain in the back ; with the second, in heart ; with the last, in pit of stomach. With the attack he has empty retching, headache, gaping, empty eructations and hot urine, and no thirst ; feels better from cold.

*Helleborus Niger* 900 (Fincke), in one prescription, cured.

*Arsenicum.*—August 24th, 1880. Mrs.—— has, with chill, gaping, stretching, numb feet, general aching worse after going up-stairs, coldness and thirst.

With the heat, nausea, headache, desire to be uncovered and increased thirst.

Sweat, with relief of symptoms.

*Arsenicum* 900 (Fincke) every two hours to-day, and after the heat if attacked to-morrow. Recovered without more medicine.

*Rhus tox.*—October 11th, 1880. Mary Ann P., aged 11 years. Has had several paroxysms of tertian intermittent, without medicine. The chill begins just before dinner. Before chill, thirst, tired and weak, can hardly stand ; with chill, coldness, shivering, face and lips blue, slight chattering of teeth, no thirst, pain in lower back, shoulders, calves and behind ears. *The cold begins in the feet.* Had taste of blood in the mouth. The chill ameliorated by covering.

The heat is burning in cheeks and hands, with red face, pain in forehead, lachrymation burning eyelids, fluent coryza ; thirst for large drinks, seldom ; nausea, and once vomiting of food. Is sleepy, but cannot sleep ; wants to be covered.

Sweat only at night ; hurts to swallow at times ; coughs more after heat ; headache during apyrexia ; urinates often in all stages.

*Rhus tox.* 900 (Fincke) every two hours.

October 14th.—No chill. There has been no subsequent return.

*Arsenicum.*—October 16th, 1880. Mr.—— had chill yesterday at 3 P. M., with pain in head and bones, lassitude, shivering and coldness, alternating with heat; thirst for small drinks of *cold* water; aching in hips and legs.

In the heat, delirious tossing; sleepiness without sleep; increased thirst; greenish bitter, violent, copious vomiting, worse after drinking.

No sweat. Tongue whitish, taste bad, worse from drinking; during apyrexia, headache, redness of face and eyes; soreness of neck and throat. Has had chills and fevers suppressed several times by allopathic treatment.

*Arsenicum,* 45 M (Fincke), three powders, to be taken two hours apart.

October 18th.—No more chills.

*Arsenicum and Nat. mur.*—November 12th, 1880. Mr.—— has had several attacks of intermittent fever during the past season treated with *Quinine,* and one, the last before this, homœopathically. Had this afternoon much gaping; heat mingled with chilliness; a feeling like sick headache in forehead and occiput.

Heat at stomach and nausea. Before the attack, increased flow of yellow urine.

*Ars.* CM (Swan), two powders, one night and morning.

14th. Coldness, with headache, at 10 A. M. Thirst at 1 P. M. Soreness about waist and back. About the same in all ways.

*Ars.* CM (Swan), two powders, one night and morning.

16th. Chill at 10 A. M., preceded by headache and nausea. With the chill, headache, much thirst, shivering, blue lips and nails, gaping, sore pain in bones and vomiting, first of food, and then water, at the end of the chill.

Heat, with sleep, red face, stupor, less thirst, woke in copious sweat of short duration. Headache in apyrexia.

He received *Nat. mur.* CM (Swan), which was not repeated after later attacks. The next chill was more severe; the second was the longest of all, but was not followed with heat, and was the last. He has since been well.

